# Protective effect of resveratrol against cadmium-induced toxicity on ovine oocyte in vitro maturation and fertilization

**DOI:** 10.1186/s40104-022-00731-1

**Published:** 2022-07-22

**Authors:** Anna Rita Piras, Federica Ariu, Alessio Maltana, Giovanni Giuseppe Leoni, Nicola Antonio Martino, Antonella Mastrorocco, Maria Elena Dell’Aquila, Luisa Bogliolo

**Affiliations:** 1 Department of Veterinary Medicine, University of Sassari, 07100 Sassari, Sardinia Italy; 2 Department of Biosciences, Biotechnologies & Biopharmaceutics, University of Bari Aldo Moro, 70125 Bari, Italy

**Keywords:** Cadmium, In vitro maturation, Oocyte, Ovine, Resveratrol

## Abstract

**Background:**

Heavy metal cadmium (Cd) is a widespread environmental contaminant with a potential toxicity that might negatively affect female reproduction and fertility. It has been reported that Cd exposure impaired the quality of oocytes and led to a defective maturation and fertilization, through oxidative stress induction. Resveratrol (Res) is a natural polyphenol with strong antioxidant properties that exhibited protective role in preventing oocyte redox homeostasis disruption and quality decline. Here, we explored whether the addition of Res to in vitro maturation (IVM) medium might act as a protection against Cd-induced toxicity on ovine oocyte maturation and fertilization. Firstly, we evaluated the effect of supplementing IVM medium with two different Res concentrations (1 and 2 μmol/L) on nuclear maturation and fertilization of oocytes matured under CdCl_2_ (2 μmol/L) exposure. Therefore, the concentration of 1 μmol/L Res was selected to analyse the effects of this compound on intracellular ROS levels, mitochondrial (mt) distribution and activity, chromatin configuration, cytoskeleton morphology, cortical granules (CGs) distribution and mRNA expression of genes associated with cellular response to oxidative stress (i.e. *SIRT1, SOD 1, GPX1, GSR, CAT*) in Cd-exposed in vitro matured oocytes.

**Results:**

We found that 1 μmol/L Res restored the reduced oocyte meiotic competence induced by Cd exposure as well as, Res sustained oocyte ability to be normally fertilized and decreased polyspermic fertilization at both tested concentrations. Moreover, we demonstrated that 1 μmol/L Res mitigated Cd-induced alterations of oocyte cytoplasmic maturation by reducing reactive oxygen species (ROS) accumulation, preventing mt dysfunction, maintaining the correct meiotic spindle and cortical F-actin assembly and the normal cortical granule distribution as well as up-regulating *SIRT1*, *SOD1* and *GPX1* genes.

**Conclusions:**

Taken together, our findings highlighted the beneficial influence exerted by Res in preventing Cd-induced disturbance of nuclear and cytoplasmic maturation and subsequent fertilization in ovine oocytes. Res treatment may help to establish defence strategies counteracting Cd-induced toxicity on the female gamete.

## Background

Anthropogenic activities and climate changes have led to a dramatic increase of chemical pollutant levels in terrestrial and aquatic ecosystems. Over recent years, a large body of studies have underlined associations between the exposure to several environmental chemicals and female reproductive disorders in humans and animals [1, 2]. Among inorganic pollutants, heavy metals represent a potential threat to reproductive health, due to the high global annual emission rate [3].

Cadmium (Cd) is considered one of the most toxic nonessential heavy metals that is widely distributed in air, water and soil. Anthropogenic Cd emissions arise from industrial processes, including the combustion of fossil fuels, waste incineration, smelting and mining, rubber processing, manufacturing of nickel-Cd batteries [3]. This metal can be found in phosphate fertilizers and it is a major component of cigarette smoke [3]. Given its low excretion from the body and its long biologic half-life (15–30 years), Cd accumulates and remains in organs and tissues over time [3]. Bioaccumulation of Cd has been found in the ovaries and follicular fluids of both humans and animals [4–7]. In the sheep, this metal was detected as the trace element with the highest age-dependent ovarian bioaccumulation [4].

Cadmium toxicity on female reproductive system has been largely explored in vivo in laboratory animal models [8–12]. Some of the adverse effect of Cd exposure include interference with the hypothalamic-pituitary-ovarian axis, reduced steroidogenesis, inhibition of follicle and oocyte development, impairment of ovulation and oocyte pick-up by the tubal epithelium together with retardation of embryo development and implantation, restricted fetal growth and pregnancy complications [13].

By using different mammalian models, previous studies highlighted the negative impact of Cd exposition on oocytes meiotic competence during in vitro maturation (IVM) and on their ability to successfully undergo in vitro fertilization (IVF) and support preimplantation embryo development. In detail, the meiotic progression of ovine, bovine, buffalo and porcine oocytes [14–18] was significantly reduced following Cd exposure during IVM. Besides, other evidences proved that in vivo and in vitro Cd exposure led to defective nuclear and cytoplasmic maturation in mouse and porcine oocytes, via the impairment of cytoskeleton assembly, spindle organization, chromosome alignment, actin polymerization, distribution of mitochondria (mt) and cortical granules (CGs) and epigenetic modifications [8, 9, 18]. The cytotoxic effect of Cd on IVM resulted in declining fertilization rates and an increased polyspermy in ovine oocytes [15] while a reduced sperm binding ability was recorded in porcine oocytes [18]. Most of the abovementioned studies were performed using Cd concentrations in the micromolar range. Previously, we evaluated the effect of Cd exposure on the fertilization rates of oocytes from juvenile and adult sheep during IVM at environmental nanomolar concentrations [4]. We found that, even at such a low Cd concentration, the IVF rate was significantly reduced and there was also a slight increase in the number of abnormally fertilized oocytes. More interestingly, our results indicated that Cd caused mt dysfunction, increased reactive oxygen species (ROS) levels and lipid peroxidation thus negatively affecting the oocyte ability to be fertilized. In agreement with our findings, the study by Zhou et al. [18], demonstrated that Cd exposure caused an excessive increase of ROS levels leading to DNA damage and apoptosis and to a defective IVM and IVF of porcine oocytes. Furthermore, in vivo acute [8] and chronic [9] Cd exposure in female mice impaired oocyte meiotic progression and decreased fertility by enhancing ROS level and apoptosis.

Based on the well-established role of oxidative stress in Cd reproductive toxicity, developing defence strategies using antioxidants is a growing field of study [19]. Resveratrol (Res; 3,4,5-trihydroxy-trans-stilbene), a phytoalexin produced by plants, is one of the most studied polyphenol with well-established antioxidant properties [20]. Several studies in various species provided evidence that Res could act as a powerful antioxidant being able to prevent the disruption of oocyte redox homeostasis and the decline of the oocyte quality and to improve oocyte IVM, IVF and subsequent embryonic development rates [21–26]. In addition, administration of Res was reported to effectively protect mouse oocytes against aging both in vivo and in vitro primarily by preventing ROS production and by improving mt function [22, 27, 28]. It has also been shown that Res enhanced the resistance of oocytes in response to sub-optimal conditions, including the exposition to toxic chemicals [29, 30], heat and hypothermic stresses [23, 31] and cryopreservation [32].

Our study aimed at exploring the effect of supplementing maturation medium with Res on IVM and IVF of ovine oocytes in vitro cultured under Cd-exposure. Given the observed positive influence of Res, its effects on the quality of Cd-exposed in vitro matured oocytes was investigated by assessing, besides chromatin configuration, intracellular ROS levels, mt distribution and activity, cytoskeleton morphology, cortical granules (CGs) distribution and mRNA expression of genes associated with cellular response to oxidative stress (i.e. *SIRT1, SOD1, GPX1, GSR, CAT*).

## Methods

### Chemicals

Unless otherwise specified, all chemicals were purchased from Sigma–Aldrich (Milan, Italy).

### Oocyte collection

Ovaries were collected from slaughtered juvenile Sarda ewes (30–40 d old) and transported to the laboratory within 3 h in phosphate-buffered saline (PBS) with penicillin (0.1 g/L) and streptomycin (0.1 g/L) at 37 °C. Cumulus oocytes complexes (COCs) were retrieved by slicing procedure in dissection medium (DM) consisting of 20 mmol/L Hepes-buffered TCM 199 supplemented with polyvinyl alcohol (0.1%, w/v) and antibiotics. COCs with two or more complete layers of compact cumulus cells and with homogeneous cytoplasm were selected for IVM.

### In vitro maturation and in vitro fertilization 

Groups of 20–25 COCs were matured in 600 μL of TCM 199 supplemented with heat-treated oestrous sheep serum (10%, OSS), pyruvate (0.36 μmol/L), cysteamine (100 μmol/L), Follicle-stimulating hormone (FSH; 1 IU/mL), Luteinizing hormone (LH; 1 IU/mL) under mineral oil, in four-well dishes (Nunc Cell Culture, Thermo Fisher Scientific, Waltham, Massachusetts, USA) in a humidified atmosphere of 5% CO_2_, at 38.5 °C for 24 h [33]. After IVM, COCs were completely denuded of granulosa cells via gentle pipetting with a fine bore glass pipette. Oocytes at the metaphase II (MII) stage were selected under a stereomicroscope (Olympus SZ-PT, Italy) for the presence of the first polar body and randomly assigned to the analyses. For IVF groups of MII oocytes from the different experimental groups were co-incubated with frozen-thawed ram spermatozoa (1 × 10^6^ spermatozoa/mL), selected by the swim-up technique in synthetic oviductal fluid (SOF, [34]) supplemented with OSS (2%), heparin (1 μg/mL), hypotaurine (1 μg/mL) for 16 h in a humidified atmosphere of 5% CO_2_ at 38.5 °C [33].

### Fertilization assessment

At the end of IVF, the presumptive zygotes were fixed in ethanol absolute with Hoechst 33258 (10 μg/mL) at 4 °C [35], mounted on microscope slides covered with cover slips and observed under an inverted fluorescence microscope (Olympus IX70, Italy). Fertilization assessment was performed as follows [4]: -normal fertilization: male and female pronuclear formation (2PN) and extrusion of two polar bodies (PBs); -polyspermic fertilization: more than two pronuclei (>2PN) and two PBs; -asynchronous fertilization: one PN and a sperm head (SH) that failed chromatin decondensation and two PBs or one PN and MII plate and two PBs; -unfertilized oocytes: MII plate and first PB.

### Determination of oocyte intracellular ROS levels

MII oocytes were incubated for 30 min in PBS with bovine serum albumin (3%, BSA) containing 10 μmol/L 2′7′- dichlorodihydrofluorescein diacetate (H_2_DCF-DA) at 38.5 °C, 5% CO_2_ in air, in order to detect the dichlorofluorescein (DCF) and localize intracellular sources of ROS [23]. After three times wash in PBS/BSA, oocytes were fixed overnight at 4 °C with 2% paraformaldehyde (PFA) solution in PBS. Then, oocytes were mounted on glass slides with 10 μg/mL Hoechst 33258 in PBS and glycerol solution (3:1, v/v) and DCF fluorescence intensity was detected by a laser-scanning confocal microscope (LSCM, Leica TCS SP5) using an argon ions laser ray at 488 nm, with 495 nm (excitation) and 520 nm (emission) filters. DCF fluorescence intensity was measured at the equatorial plane using the LAS AF Lite 170 image analysis software package (Leica Microsystems GmbH, Wetzlar, Germany) on the whole cytoplasmic areas and the average fluorescence intensity per unit area was determined. The intensities of signals were expressed as arbitrary units (A.U).

### Evaluation of mitochondrial distribution and activity

MII oocytes were stained with MitoTracker Orange CMTM Ros (Molecular Probes, Inc., Eugene, OR, USA) in order to detect active mt distribution and mt activity. Briefly, oocytes were incubated for 30 min, at 38.5 °C, 5% CO_2_ in air, in PBS/BSA containing 280 nmol/L MitoTracker Orange CMTM Ros, Then, oocytes were mounted on glass slides with 10 μg/mL Hoechst 33258 in PBS and glycerol solution and imaged under TCS SP5 LSCM. A helium/neon laser ray at 543 nm, equipped with 551 nm (excitation) and 576 nm (emission) filters, was used to point out the MitoTracker Orange CMTM Ros. LSCM settings were kept constant for all experiments.

In each individual oocyte, MitoTracker fluorescence intensity was measured on the section corresponding to the oocyte equatorial plane, as described for ROS evaluation. The intensities of signals were expressed as A.U.

A homogeneous mt distribution, with small granulations spread throughout the cytoplasm was considered as the normal mt distribution pattern [36]. The percentage of oocytes with normal and abnormal mt distribution was calculated in each group.

### Immunofluorescence detection of cytoskeletal structures (meiotic spindle, cortical F-actin)

MII oocytes were fixed in microtubule-stabilising buffer (100 mmol/L PIPES, 5 mmol/L MgCl_2_, 2.5 mol/L EGTA, 2% formaldehyde, 0.1% Triton X-100, 1 mol/L taxol, 10 U/mL aprotinin and 50% deuterium oxide) for 1 h at 37 °C and stored in blocking solution (0.2% sodium azide, 2% normal goat serum, 1% BSA, 0.1 mol/L glycine and 0.1% Triton X-100 in PBS) at 4 °C until processing [37]. Samples were incubated overnight at 4 °C with a mixture of mouse monoclonal anti α-tubulin (dilution 1:1000), and mouse monoclonal anti β-tubulin (dilution 1:100) antibodies followed by incubation with donkey anti-mouse fluorescein isothiocyanate-conjugated antibodies (FITC-Alexa Fluor 488, dilution 1:100; Life Technologies, Invitrogen, Carlsbad, California, USA) combined with rhodamine-phalloidin (dilution 1:150; Invitrogen, Carlsbad, CA) for 1 h at room temperature. DNA was stained with Hoechst 33258 (10 μg/mL). Images of chromatin, meiotic spindles, and cortical F-actin were acquired by LSCM equipped by Ar/He/Ne lasers, using a 40 × oil objective. Hoechst 33258, FITC and rhodamine-phalloidin were excited at 358 nm, 488 nm, and 551 nm wavelengths, and emissions were detected at 461 nm, 550 nm, 595 nm, respectively.

Oocytes were scanned through the Z-axis and the images of the F-actin were recorded on the section corresponding to the equatorial plane of the cell. Oocytes were classified having a normal (symmetrical barrel-shaped) and abnormal (disorganized, clumped or dispersed elements) meiotic spindle and a normal (aligned chromosomes at the equatorial plate) or abnormal (misaligned or dispersed chromosomes) chromatin [38, 39]; a normal (an evenly stained layer of F-actin band immediately beneath the plasma membrane) or abnormal (irregular, discontinuous F-actin band) cortical F-actin distribution [38, 40].

The proportion of oocytes with normal and abnormal spindle, chromatin and cortical F-actin configuration was counted in each group.

### Assessment of CGs distribution

Analysis of CGs was performed according to the methodology of Hosseini et al. [41]. MII oocytes were incubated with pronase (0.5%) at 37 °C to remove the zona pellucida and fixed with PFA (2%) for 30 min at room temperature. After fixation, oocytes were permeabilized in PBS with Triton X-100 (0.1%) for 5 min, and then incubated in PBS with lectin peanut agglutinin (100 μg/mL, PNA) Alexa Fluor 488-conjugated (Molecular probes, Invitrogen) for 30 min at 37 °C. After three washes in PBS with BSA (0.3%), glycine (100-mmol/L) and Triton X-100 (0.01%), DNA was stained with Hoechst 33258 (10 μg/mL) and oocytes were mounted on glass slides overlaid with a coverslip. The distribution of CGs was examined at the equatorial plane of the oocytes by LSCM (490-nm excitation wavelength). Cortical granules localized in oocyte cortical cytoplasm were considered as a normal distribution pattern [37, 42]. The proportion of oocytes with normal and abnormal CGs distribution was recorded.

### RNA extraction and quantitative real-time RT-PCR

Three groups of fifteen MII oocytes each were analysed for each experimental conditions. Oocytes were snap frozen in liquid nitrogen and stored at − 80 °C until further analysis. Total RNA was isolated from oocytes with RNeasy Micro Kit (Qiagen, Hilden, Germany) following manufacturer’s instructions. Extracted RNA was treated with DNase I to exclude any potential genomic DNA contamination. The RNA isolated from oocytes was entirely and immediately used for reverse transcription-polymerase chain reaction (RT-PCR). The High Capacity Complementary DNA (cDNA) Reverse Transcription kit (Life Technologies, Monza, Italy) was used to convert RNA to cDNA. Briefly, the total RNA of each sample was added to 2 μL 10× RT buffer, 0.8 μL 25× dNTP mix, 2 μL RT random primers, 1 μL M-MLV RT, 1 μL RNase inhibitor, and nuclease-free H_2_O for a total volume of 20 μL and incubated at 10 °C for 10 min, then at 37 °C for 120 min, and finally at 85 °C for 5 min. The relative quantification of the transcripts was carried out by Real Time RT-PCR with the StepOne instrument (Applied Biosystems, Foster City, CA, USA) using specific primers for each gene (Table 1). RT-PCR was performed in 20 μL reaction volume containing: 10 μL PowerUp SYBR Green PCR Master Mix (Applied Biosystems, 2×), 1 μL of each primer (200 nmol/L), 1 μL of cDNA and nuclease-free water up to 20 μL. The temperature protocol consisted in 2′ at 95 °C followed by 40 cycles at 94 °C (45 s), 60 °C (45 s) and 72 °C (45 s). A melting curve was finally performed to detect PCR specificity. Relative quantification was performed by using the 2^-ΔΔCt^ method according to Livak and Schmittgen [43] using the β-actin as housekeeping gene.

**Table 1:** Information of primers used in RT-qPCR

Symbol	Gene name	Accession ID	Primer Seq (5′ to 3′)	Tm, °C
*SIRT1*	Sirtuin 1	XM_012125645.2	AGTTGGTGATGGACAGGGAG CGCTAGTTCAGTTCAGTCGC	59
*SOD1*	Superoxide dismutase 1	NM_001145185	GGCAATGTGAAGGCTGACAA TGCCCAAGTCATCTGGTCTT	59
*GPX1*	Glutathione peroxidase 1	GAAI01007125	ACCCAGATGAATGACCTGCA TCGGACGTACTTCAGGCAAT	59
*GSR*	Glutathione-disulfide reductase	XM_015104590	CTGCCCTGGGTTCTAAGACA ACCTGGGAGTACTTCAGCAC	59
*CAT*	Catalase	GQ421282	ACGCCTGTGTGAGAACATTG AGCCATACTCAGGATGGACA	59
*ACTB*	Actin B	NM_001009784	CCCTGGAGAAGAGCTACGAG TAGTTTCGTGAATGCCGCAG	59

### Experimental design

#### Exp. 1

Firstly, our aim was to evaluate the effects of supplementing IVM medium with two different Res concentrations on the nuclear maturation and fertilization rates of oocytes matured under cadmium (Cadmium chloride, CdCl_2_) exposure. To this end, COCs were randomly divided in four groups and cultured in IVM medium supplemented with 0 μmol/L CdCl_2_ (control, Ctr-group), 2 μmol/L CdCl_2_ (Cd-group), 2 μmol/L CdCl_2_ + 1 μmol/L Res (Cd Res 1-group) and 2 μmol/L CdCl_2_ + 2 μmol/L Res (Cd Res 2-group).

After IVM, nuclear maturation was assessed by the presence of the first PB. Oocytes showing the first PB from the different experimental groups underwent IVF, followed by the evaluation of the percentage of normally and abnormally fertilized oocytes.

The concentration of 2 μmol/L Cd was selected because it represents an intermediate concentration compared with those previously used in in vitro Cd-toxicity studies in various animal models [14–16]. In addition, in a previous report [15], we observed that the exposure of ovine oocytes to 2 μmol/L Cd reduced the maturation and fertilization rate and increased polyspermy whereas higher concentrations exhibited lethal toxicity.

CdCl_2_ was dissolved in PBS (10 mmol/L CdCl_2_ stock solution) and diluted in TCM199 medium to a final concentration of 2 μmol/L immediately before culture.

#### Exp. 2

Based on the results of Exp. 1, we explored the effect of Res supplementation (1 μmol/L) during IVM on the quality of Cd-exposed in vitro matured oocyte by evaluating intracellular ROS levels, the mt distribution and activity, the chromatin configuration and morphology of meiotic spindle and cortical F-actin, the distribution of CGs, as well as, the mRNA expression of *SIRT1, SOD1, GPX1, GSR* and *CAT* genes.

### Statistical analysis

All the experiments in the present study were replicated at least three times. Statistical analyses were performed using STATA\IC 11.28 (StataCorp LP, Lakeway Drive, college station, TX, USA).

Categorical data on IVM, IVF, mt and CGs distribution, chromatin and cytoskeleton morphology were analyzed by Chi-square test or the Fisher’s exact post-hoc test when appropriate.

The Shapiro-Wilk test was used to verify the normal distribution of data on intracellular ROS level, mt activity and gene expression. Parametric analysis of variance (ANOVA) with Bonferroni correction was made to compare normally distributed data. A post-hoc Tukey test was used to explore difference among experimental groups when data were statistically different. When not normally distributed, a non-parametric Kruskal-Wallis test was used.

Differences with *P* < 0.05 were considered statistically significant.

## Results

### Exp. 1

#### Res counteracted Cd-induced reduction of oocyte maturation and fertilization

**Fig. 1:**
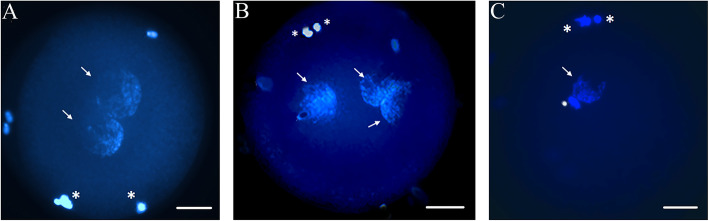
Representative photomicrographs of nuclear configuration of juvenile sheep oocytes 16 h post-in vitro fertilization in Cd-exposed oocytes. **A** zygote with two PNs and two PBs (normal fertilization); **B** polyspermic fertilized oocyte with three PNs and two PBs and **C** asynchronous fertilized oocyte with one PN, an intact sperm head (SH) and two PBs. PN: arrow; PB: asterisk; SH: dot Scale bar = 25 μm

The results of Exp. 1 are summarized in Table 2. The incidence of polar body extrusion after IVM was lower in the Cd-group compared to the Ctr-group (*P* < 0.05). Supplementation with 1 μmol/L Res enhanced oocyte maturation rate compared to the Cd-group (*P* < 0.05). No differences were recorded among the Cd Res 2-group and other groups. Cd exposure during IVM reduced the normal fertilization rate (*P* < 0.05) and increased the percentage of oocytes showing more than 2PN (*P* < 0.01) compared to the Ctr-group. Res, at both tested concentrations, enhanced the occurrence of 2PN formation (*P* < 0.05) and decreased the polyspermic fertilization compared to the Cd-group *(P* < 0.01). In Fig. 1 representative micrographs of normal (Fig. 1A), polyspermic (Fig. 1B) and asynchronous fertilization (Fig. 1C) after IVF of oocytes matured in presence of Cd.

**Table 2:** Effect of Res supplementation during IVM on meiotic maturation and fertilization of Cd-exposed oocytes

Groups	N° of oocytes	N° of MII oocytes	N° of fertilized oocytes, %			
			Normal (2PNs)	Polyspermy (> 2PNs)	Asynchronous	Unfertilized
Ctr	120	105 (87.5%)^a^	59 (56.2%)^a^	23 (21.9%)^b^	6 (4.8%)	17 (16.2%)
Cd	146	106 (72.6%)^b^	39 (36.8%)^b^	38 (35.8%)^c^	7 (6.6%)	22 (20.7%)
Cd-Res 1	105	88 (83.8%)^a^	47 (53.4%)^a^	16 (18.2%)^b^	7 (7.9%)	18 (20.4%)
Cd-Res 2	102	80 (78.4%)^ab^	41 (51.2%)^a^	13 (16.2%)^b^	6 (7.5%)	20 (25.0%)

The rate of fertilization was calculated from the number of MII oocytes. Values with different superscripts (a, b, and c) in the same column are significantly different (a vs. b: *P* < 0.05; b vs. c: *P* < 0.01)

### Exp. 2

#### Res allowed redox equilibrium recover in Cd-exposed oocytes

**Fig. 2:**
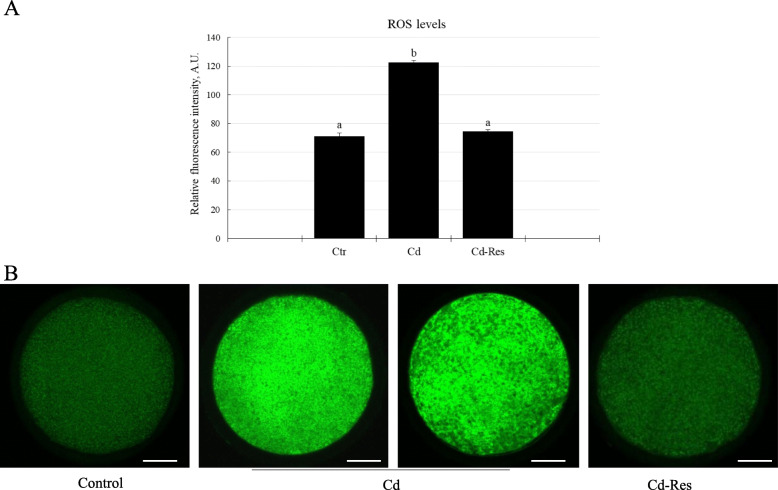
Effect of Res on the intracellular ROS levels in Cd-exposed oocytes. **A** Quantification of fluorescence intensity after incubation with the DCHFDA probe. **B** Representative LSCM photomicrographs of ROS levels (green) in oocytes from Ctr, Cd and Cd-Res groups. The data are expressed as mean ± standard error. A.U: arbitrary units. Values with different superscripts (a, b) are significantly different (*P* < 0.01). Scale bar = 25 μm

As shown in Fig. 2, DCF fluorescence intensity indicating intracellular ROS levels was significantly higher in the Cd-group than the Ctr-group (122.4 ± 1.5 A.U, *n* = 70 vs. 71.1 ± 2.3 A.U, *n* = 75*; P* < 0.05; Fig. 2A and B). On the contrary, Res supplementation reduced ROS levels (74.4 ± 1.3 A.U),

*n* = 73; *P* < 0.05, Fig. 2 A and B) as compared to the Cd-group, reaching a value comparable to that of Ctr oocytes.

#### Res restored mitochondria distribution pattern and basal activity level in Cd-exposed oocytes

**Fig. 3:**
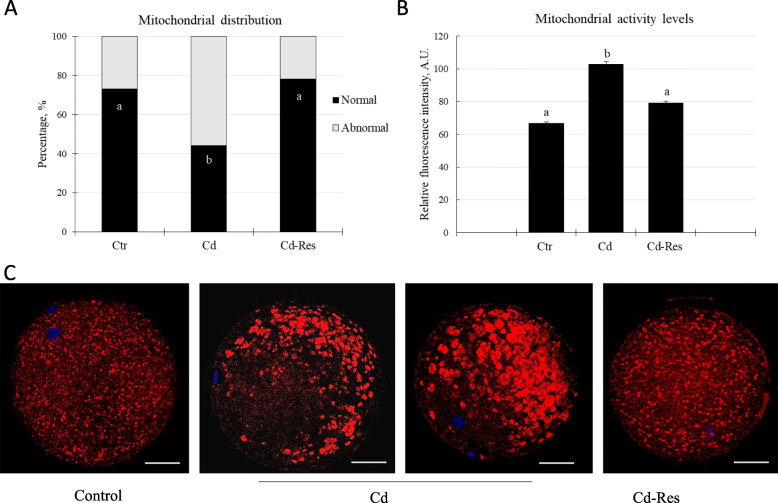
Effect of Res on the distribution and activity of mitochondria in Cd-exposed oocytes. Graphs showing **A** the percentage of oocytes with normal and abnormal mt distribution pattern; **B** quantification of fluorescence intensity after incubation with MitoTracker Orange; **C** Representative LSCM photomicrographs of mt distribution (red) in oocytes from Ctr, Cd and Cd-Res groups. The data are expressed as mean ± standard error. A.U: arbitrary units. Values with different superscripts (a, b) are significantly different (*P* < 0.01). Scale bar = 25 μm

After IVM, 73.1% of oocytes of the Ctr-group (*n* = 38/52) showed homogeneous mt distribution pattern with small granulations spread throughout the cytoplasm (normal distribution, Fig. 3 A and C). This pattern was severely altered in oocytes of the Cd-group, which exhibited mt disposition pattern with medium/large granulations located in the specific cytoplasmic area (abnormal distribution, Fig. 3C). The proportion of oocytes with normal mt distribution in the Cd-group was lower (44.1%; *n* = 26/59, *P* < 0.01) than that of the Ctr-group (Fig. 3A). Res treatment restored the rate of normal mt distribution (78.3%, *n* = 47/60; Fig. 3 A and C) with a value higher than in the Cd-group (*P* < 0.01) and similar to the Ctr-group.

Mitochondria activity was higher in the Cd-group than the Ctr-group (103.1 ± 1.4 A.U; *n* = 59 vs. 67 ± 0.6 A.U, *n* = 52, *P* < 0.01; Fig. 3B). Res supplementation decreased mt activity (79.3 ± 1.1 A.U, *n* = 60; *P* < 0.01) to a level similar to that of Ctr oocytes and lower than that of the Cd-group (Fig. 3B).

#### Res re-established spindle and cytoplasmic cytoskeletal structures in Cd-exposed oocytes

**Fig. 4:**
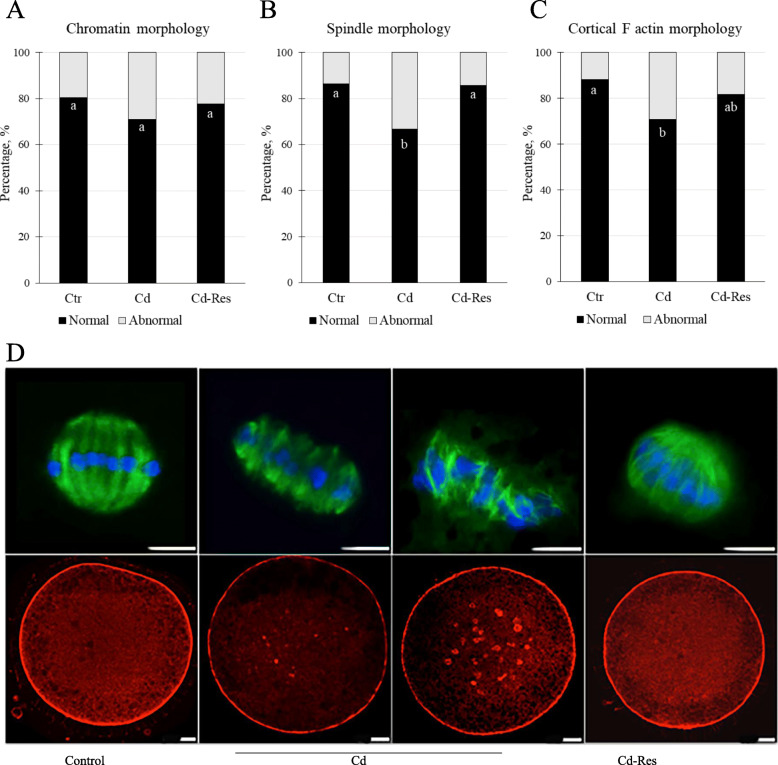
Effect of Res on chromatin and cytoskeleton morphology in Cd-exposed oocytes. **A** Graph showing the percentage of oocytes with normal and abnormal chromatin, meiotic spindle and F-actin configuration. Representative LSCM images of (**B**) chromosomes (blue) and meiotic spindle (green) and (**C**) actin filaments (red) in oocytes of Ctr, Cd and Cd-Res groups. Values with different superscripts (a, b) are significantly different (*P* < 0.05). Scale bar = 10 μm

Figure 4 reports the results of LSCM analysis of chromatin, spindle and cortical F-actin of oocytes from Ctr, Cd and Cd-Res groups. The organization of chromosomes did not differ among groups (Fig. 4 A and B). The meiotic spindle in oocytes from the Ctr group had a classical barrel-shape structure (Fig. 4B). The rate of oocytes with normal meiotic spindle structure decreased in the Cd group (66.7%, *n* = 32/48) compared to the rate in the Ctr group (86.3%, *n* = 44/51; *P* < 0.05, Fig. 4A). Res supplementation increased the percentage of oocytes with normal spindle structures (85.7%, *n* = 42/49, *P* < 0.05) compared to those of the Cd-group reaching a value comparable to the level in the Ctr-group (Fig. 4A). Concerning the cortical F-actin network, oocytes in the Ctr-group displayed a normal one beneath the oolemma (Fig. 4C). Instead, actin filaments were abnormally distributed in Cd-group oocytes which displayed irregular, discontinuous F-actin staining in the cortical area or spotted staining within the oocyte cytoplasm (Fig. 4C). The percentage of oocytes with a normal pattern of F-actin organization was lower in the Cd-group (68.7%, *n* = 33/48) as compared to the Ctr-group (88.2%, *n* = 45/51; *P* < 0.05, Fig. 4A). Res treatment partially restored the normal F-actin configuration with a rate (81.6%, *n* = 40/49; Fig. 4A) not statistically different from the Cd-group but similar to Ctr oocytes.

#### Res recovered normal CG distribution in Cd-exposed oocytes

**Fig. 5:**
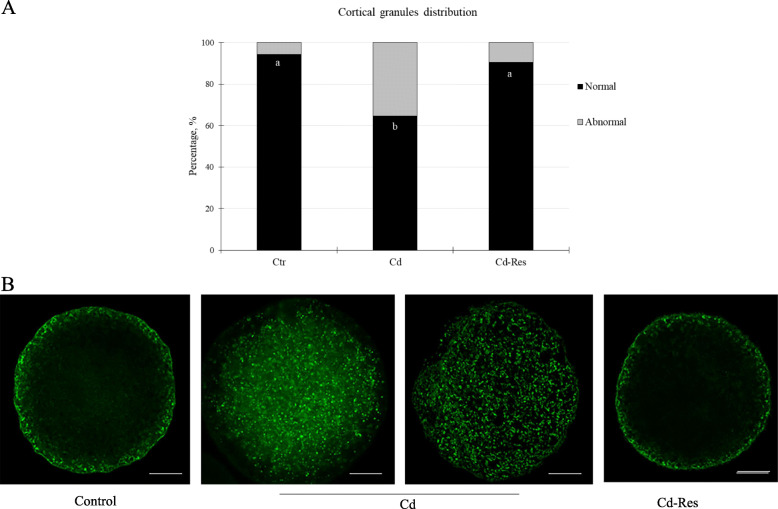
Effect of resveratrol on CGs distribution in Cd-exposed oocytes. **A** Graph showing the percentage of oocytes with normal and abnormal CGs distribution and (**B**) representative images of CGs (green) localization in Ctr, Cd and Cd-Res oocytes. Values with different superscripts (a, b) are significantly different (*P* < 0.05). Scale bar = 25 μm

Most of Ctr oocytes (94.4%, *n* = 34/36, Fig. 5A) displayed the CGs localized beneath the oolemma (normal distribution, Fig. 5B). On the contrary, the Cd-group showed an abnormal distribution of CGs which were diffused through the oocyte cytoplasm (Fig. 5B). The percentage of oocytes with a normal pattern of CGs distribution was lower in the Cd-group (64.7%, *n* = 22/34) than in the Ctr-group (*P* < 0.01, Fig. 5A). Res supplementation increased the rate of oocytes with normal distribution of CGs (90.6%, *n* = 29/32; Fig. 5A and B) as compared to the Cd-group reaching a value similar to the one in the Ctr-group.

#### Res re-established gene expression in Cd-exposed oocytes

**Fig. 6:**
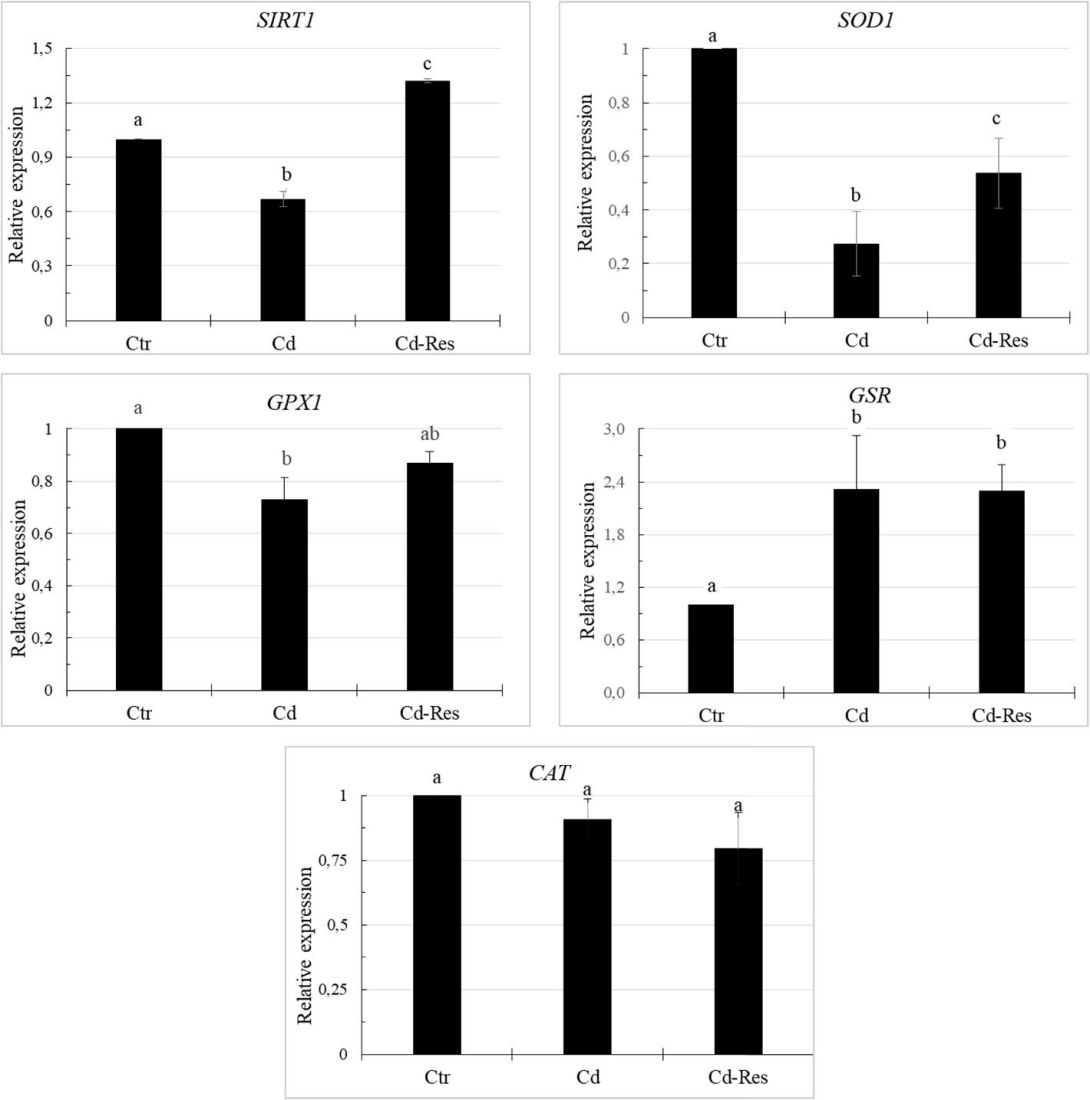
Effect of Res on relative expression of *SIRT1, SOD1, GPX1, GSR* and *CAT* genes in Ctr, Cd and Cd-Res oocytes. The data are expressed as mean ± standard deviation. Values with different superscripts (a, b, c) are significantly different (*P* < 0.05)

In Fig. 6, the expression analysis of specific genes related to oocyte response to oxidative stress is shown. In the Cd-group the expression of *SIRT1, SOD1* and *GPX1* genes significantly decreased while *GSR* gene expression was up-regulated as related to the control (*P* < 0.05). Res treatment increased the transcript levels of *SIRT1* when compared to the Ctr and Cd groups (*P* < 0.05) and up-regulated the expression of *SOD1* as related to the Cd-group (*P* < 0.05). The expression of *GPX1* gene in the Cd-Res group reached a value comparable to the level in the Ctr-group. In the Cd-Res group the expression of *GSR* gene was similar as compared to that of the Cd-group and higher (*P* < 0.05) than the Ctr-group. The relative gene expression levels of *CAT* gene did not differ among groups.

## Discussion

The induction of oxidative stress has been indicated as one of the major factors that may be responsible for Cd-induced deterioration of oocyte quality and function [9]. Antioxidant administration may act as an effective strategy to counteract Cd toxicity on the female gamete. In this sense, the beneficial role exerted by melatonin and quercetin in preventing Cd-induced oxidative stress in rat ovaries has been recently highlighted [44, 45]. Actually, only one study investigated the potential of in vitro treatment with antioxidants against Cd-induced adverse effects on mammalian oocyte quality and meiotic competence demonstrating that glutathione supplementation during IVM was effective to recover the Cd-induced meiotic failure in porcine oocytes through suppressing excessive ROS levels [18]. Therefore, the present research might provide additional contribution to set up antioxidant protective strategies aiming at coping with Cd-induced toxicity on female gametes. We investigated the potential beneficial effect of Res as it acts as a natural antioxidant against the toxic effects exerted by Cd exposure on ovine oocytes in vitro maturation and fertilization.

In line with previous data from other animal models [14, 16, 18], our results confirmed that Cd exposure caused a defective nuclear and cytoplasmic maturation and impaired normal fertilization in ovine oocytes. Furthermore, Cd-exposed oocytes displayed excessive ROS production, mt dysfunctions, disturbances in cytoskeleton assembly and CGs distribution and alterations of oxidative-stress related gene expression. More importantly, the main research findings showed that Res effectively mitigated Cd-induced alterations of oocyte meiotic and cytoplasmic maturation and restored normal fertilization.

Several studies carried out on various animal species provided evidences that Res was helpful for protecting meiotic and developmental competence of oocytes under sub-optimal conditions [22, 27, 46]. Although the optimal concentration of Res varied between species and experimental conditions [21], the overall results indicated that Res addition to culture medium at doses ranging between 0.5 μmol/L to 2 μmol/L allowed to achieve the best results [22, 29, 31, 32, 47]. Therefore, we explored the effect on meiotic maturation and fertilization of Cd-exposed oocytes through supplementing the IVM medium with 1 and 2 μmol/L Res concentrations. We found that 1 μmol/L Res treatment was effective to recover the reduced oocyte meiotic competence induced by Cd exposure. Moreover, at both tested concentrations, Res preserved the oocyte ability to be normally fertilized with corresponding decrease of polyspermic fertilization.

These results suggested that 1 μmol/L Res was able to preserve oocytes against Cd-induced quality and function deterioration, which causes defective maturation and fertilization.

Subsequently, in order to explore the biological mechanisms underlying Res effects against Cd-induced oocyte toxicity, a series of correlated and complementary cellular and molecular parameters, that can be influenced by oxidative stress, were evaluated in in vitro matured Cd-exposed oocytes co-treated with 1 μmol/L Res.

First, we demonstrated that Res was able to scavenge ROS overproduction induced by Cd-exposure, allowing redox equilibrium recover. It is widely described that the protective effect of Res in improving oocyte quality and developmental competence is strictly related to its ability to eliminate excessive ROS and to inhibit early apoptosis [21, 23, 27, 29, 30, 47, 48].

Cd-induced excessive ROS can cause mt dysfunction with a consequent increase of mt ROS and cellular oxidative damage [49]. Mitochondria play a vital function during oocyte maturation, fertilization as they provide energy and regulate redox homeostasis [50]. Correct mt functioning and distribution are critical for proper oocyte maturation and fertilization [36]. Cd-induced mt dysfunction have been linked to oocyte meiotic arrest and fertilization failure in porcine [18], mouse [8, 12] and ovine [4] oocytes. In our study, Cd caused mt location changes within the oocytes cytoplasm as well as mt over-activity. These events might have generated structural modifications in the oocyte bioenergetics status and triggered oxidative stress conditions. In line with these results, we previously demonstrated that exposure to nanomolar Cd concentration altered the bioenergetics/oxidative status of ovine oocytes by increasing mt activity and ROS production [4]. We suggest that mt over-activity might represent a response to changes in oocytes energy demand following oocytes exposure to Cd as a way to activate defence mechanisms against Cd-induced redox unbalance and it may be expressed as an early event that anticipates mt function loss. Res treatment was effective to prevent mt translocation and to regulate mt activity leading to recover condition similar to controls. Therefore, our data indicated that Res, through ROS reduction, avoided inadequate translocation of mt and disturbances of mt function which, in turn, might have promoted proper maturation and fertilization conditions.

An oocyte component that can be damaged by elevated ROS levels is the cytoskeleton [51]. The organization and dynamic of spindle microtubules and actin filaments are a crucial requisites to drive meiotic progression, polar body extrusion and to establish the cortical polarity during oocyte maturation [52]. Likewise, cytoskeletons components, plays an important role in the distribution of some organelles as mitochondria [52]. Our findings revealed that Cd exposure perturbed the meiotic spindle assembly and actin filaments on the oocyte plasma membrane, but did not altered the chromosome condensation or alignment at the MII stage. Instead, Res treatments mitigated Cd-induced meiotic spindle disturbance and partially restored the distribution of F-actin in the oocyte cortex. Thus, we postulate that the protective effect of Res against Cd-induced ROS overproduction and mt dysfunction might be reflected in the maintenance of the correct cytoskeleton assembly with subsequent normal rate of nuclear maturation [53].

Our study also revealed that Res ameliorated the fertilization capability of Cd exposed oocytes and prevented polyspermic fertilization. The distribution of CGs beneath the oolemma in matured oocytes and exocytosis of their content after fertilization are crucial steps to block polyspermic fertilization [54]. In relation to this, we demonstrated that Cd exposure during IVM disturbed CGs redistribution in the cortex of the oocyte and that Res ensured normal dynamics and localization of CGs, therefore avoiding polyspermic fertilization as a result. According to our findings, Res improved the correct CGs distribution before in vitro fertilization as previously observed in bovine [55] and porcine oocytes [46]. The protective effect of Res on CGs migration and fertilization might be obtained through the maintenance of proper mt function and microfilament dynamic, which are involved in the process of CGs migration and exocytosis [54].

Finally, transcriptional experiments were addressed to measure the mRNA levels of *SIRT1* and the antioxidant genes *SOD1, GPX1, GSR* and *CAT*, known to be involved in cellular response to oxidative stress [56]. Specifically, we detected lower expression of *SIRT1*, *SOD1* and *GPX1* transcripts in Cd-exposed oocytes while the use of Res reversed this effect by enhancing the mRNA transcript levels of these genes.

Although resveratrol is able to directly scavenge a variety of free radicals, its main antioxidant effect is more properly due to its regulation of redox genes [20]. SOD1 is one of the three superoxide dismutases known to catalyse the dismutation of superoxide (O_2_^−^) to hydrogen peroxide (H_2_O_2_) and O_2_; at the same time, GPX1 and CAT catalyse the rapid decomposition of H_2_O_2_ while GSR is involved in the glutathione-dependent antioxidant system by reducing oxidized glutathione [57].

Previous studies showed that supplementing IVM medium with Res induced the up-regulation of various antioxidant genes including *SOD1*, *GPX1* and *CAT* in oocytes from aged mice [22] and in bovine and porcine oocytes [58, 59]. Similarly, in the present study, Res treatment determined the upregulation of these genes, thus supporting the notion that Res might have counteracted Cd-induced oxidative stress through increasing the expression of antioxidants genes.

A further effect of Res-induced response against Cd toxicity might be linked to *SIRT1* overexpression. The main pathways of Res action is mediated by SIRT1, a NAD-dependent deacetylase which acts as regulator of the redox state in the oocytes [56], also involved in mitochondrial biogenesis and degradation [60]. Cd induced suppression of SIRT1 signalling has been demonstrated in several cell lines determining oxidative stress, apoptosis, mt and metabolic dysfunctions [61]. By up-regulating *SIRT1*, Res increased the expression of *SOD1*, *GPX1* and *CAT* in cultured cells [62]. Our data suggested that Res might have counteracted Cd-toxicity by up-regulating *SIRT1* contributing to overexpression of the antioxidant genes such as, *SOD1* and *GPX1*. We can also hypothesize that Res might have stimulated SIRT1 mediated mt turnover in Cd-exposed oocytes [63, 64]. This aspect requires further investigations. Interestingly, the expression level of the *SIRT1* gene was significantly higher in oocytes treated with Res compared to Ctr oocytes, indicating its effect in boosting oocyte antioxidant defence. This evidence suggests that SIRT1 pathway activation may play a key role underlying the Res induced response against Cd-induced toxicity on oocyte maturation.

## Conclusions

In summary, our results demonstrated that Res supplementation during IVM of Cd-exposed ovine oocytes can reverse the Cd-induced oxidative stress by directly scavenging ROS and/or promoting the activity of molecular targets involved in the regulation of the cellular response to oxidative stress. The ameliorative influence of Res was reflected by improvement of mitochondrial function, cytoskeleton morphology, CGs distribution and up-regulating *SIRT1, SOD1, GPX1*, and ultimately by enhancement of oocyte maturation and fertilization. To thoroughly explore the underlying mechanisms of Res protection, further studies should focus on one hand on assessing SIRT1 pathway modulators and antioxidant enzymes activity and on the other hand on evaluating Res effects on cleavage and blastocyst formation during IVM in Cd-exposed oocytes. Moreover, in vivo studies are obviously warranted to validate effectiveness and safety of Res to protect oocytes against Cd-induced toxicity.

## Data Availability

All data generated or analysed during this study are included in this published article.

## Abbreviations

Cd: Cadmium
Res: Resveratrol
IVM: In vitro maturation
CdCl_2_: Cadmium chloride
Mt: Mitochondria
ROS: Reactive oxygen species
CGs: Cortical granules
SIRT1: Sirtuin 1
SOD1: Superoxide dismutase 1
GPX1: Glutathione peroxidase 1
GSR: Glutathione-disulphide reductase
CAT: Catalase
IVF: In vitro fertilization
PBS: Phosphate-buffered saline
COCs: Cumulus-oocyte complexes
DM: Dissection medium
OSS: Oestrous sheep serum
FSH: Follicle-stimulating hormone
LH: Luteinizing hormone
MII: metaphase II
SOF: Synthetic oviductal fluid
PN: Pronucleus
PB: Polar body
SH: Sperm head
H_2_DCF-DA: 2′7′- dichlorodihydrofluorescein diacetate
BSA: Bovine serum albumin
PFA: Paraformaldehyde
DCF: Dichlorofluorescein
LSCM: Laser-scanning confocal microscope
A.U: Arbitrary unit
PNA: Lectin peanut agglutinin
RT-PCR: Reverse transcription-polymerase chain reaction
cDNA: Complementary DNA
ANOVA: Parametric analysis of variance
